# Transition-Metal Dichalcogenides in Electrochemical Batteries and Solar Cells

**DOI:** 10.3390/mi14030691

**Published:** 2023-03-21

**Authors:** Mohammad Bagher Askari, Parisa Salarizadeh, Payam Veisi, Elham Samiei, Homa Saeidfirozeh, Mohammad Taghi Tourchi Moghadam, Antonio Di Bartolomeo

**Affiliations:** 1 Department of Semiconductor, Institute of Science and High Technology and Environmental Sciences, Graduate University of Advanced Technology, Kerman P.O. Box 7631818356, Iran; 2 High-Temperature Fuel Cell Research Department, Vali-e-Asr University of Rafsanjan, Rafsanjan P.O. Box 7718897111, Iran; 3 Applied Chemistry Research Laboratory, Department of Chemistry, Faculty of Science, University of Zanjan, Zanjan P.O. Box 45195-313, Iran; 4 Department of Photonics, Institute of Science and High Technology and Environmental Sciences, Graduate University of Advanced Technology, Kerman P.O. Box 7631818356, Iran; 5 J. Heyrovský Institute of Physical Chemistry, Czech Academy of Sciences, Dolejškova 3, CZ 18223 Prague, Czech Republic; 6 Department of Physics, Faculty of Science, University of Guilan, Rasht P.O. Box 41335-1914, Iran; 7 Department of Physics “E. R. Caianiello”, University of Salerno, Fisciano, 84084 Salerno, Italy

**Keywords:** transition metal dichalcogenides, electrochemical batteries, supercapacitors, solar cells

## Abstract

The advent of new nanomaterials has resulted in dramatic developments in the field of energy production and storage. Due to their unique structure and properties, transition metal dichalcogenides (TMDs) are the most promising from the list of materials recently introduced in the field. The amazing progress in the use TMDs for energy storage and production inspired us to review the recent research on TMD-based catalysts and electrode materials. In this report, we examine TMDs in a variety of electrochemical batteries and solar cells with special focus on MoS_2_ as the most studied and used TMD material.

## 1. Introduction

Over the past decade, transition metal dichalcogenides (TMDs) have attracted widespread scientific interest due to their special and often layer-tunable chemical, thermal, mechanical, electronic, magnetic, and optical properties [1,2,3,4,5]. TMDs have the chemical formula MX_2_, where M is a transition metal and X is a chalcogen from the group VI-A. In general, TMDs with transition metals from 4B–7B groups have a layered structure, while TMDs with transition metals from 8B–10B groups, such as pyrite, have a non-layered structure. The atoms in the MX_2_ layered structure are in polytype mode, with one transition metal atom surrounded by six chalcogen atoms [6].

Following the discovery of strong photoluminescence in MoS_2_ monolayers [7] and the demonstration of the first transistor [8], significant studies were performed on the application of TMDs in fields such as energy storage [9,10], energy production [11,12,13] electronics [14,15,16], and optoelectronics [17,18].

Molybdenite as the first structure of TMDs was determined in 1923 by Linus Pauling [19]. Over the following 40 years, around 40 TMDs with a layered structure became known [20]. However, the first attempt to produce ultrathin MoS_2_ layers by use of adhesive tapes in 1963 was performed by Robert Frindt [21]. Later, in 1986 the production of monolayer MoS_2_ suspensions was achieved [22]. The discovery of WS_2_ nanotubes and nested particles [23], followed by the synthesis of MoS_2_ nanotubes and fullerenes, further boosted this field [24]. After 2004, the rapid growth of graphene-based research sparked the interest in layered materials that, as graphene, could be exfoliated or produced in the monolayer form and led to the development of techniques well suited for new studies on two-dimensional (2D) materials.

The use of two-dimensional materials in various fields of science is of great interest [25,26]. The structural similarity to graphite and graphene is combined with specific and very special properties in TMDs. In general, the three-atom thick unit cell of TMDs is formed by a layer of transition metal atoms (e.g., Mo, W, Ta, etc.) sandwiched between two layers of chalcogen atoms (e.g., S, Se, Te). Due to the weak bonds between adjacent layers, TMDs can be thinned down to single layers [27,28]. The weak interlayer adhesion is caused by the presence of a gap or voids between the layers and is still a matter of debate in the scientific community [29,30].

A van der Waals gap between the layers, affecting the electronic and optical properties of the material, is supported by experimental evidence, such as scanning tunneling microscopy and X-ray diffraction studies, which have shown a clear separation between the layers in TMDs [30]. On the other hand, some studies have suggested that there may be voids rather than a gap between the layers in TMDs. These voids exist between the chalcogenides in the interfacial/interlayer region and are thought to be responsible for the weakening of the weak forces in the interlayer region. This view is also supported by experimental evidence, such as transmission electron microscopy studies, which have shown a lack of a clear separation between the layers in some TMDs [31].

It is important to note that the occurrence of a specific type of force between the layers depends on the nature of the transition metal chalcogenide system. The intercalation of foreign species, such as Li, can modify the interfacial geometry of TMDs and increase the distance between two consecutive monolayers, which in turn can tune the electronic and optical properties of the material. However, as noted in several studies, intercalation can also result in the loss of the pristine semiconducting properties of TMDs due to structural changes in the host materials [32]. While the debate over the presence of a gap or voids between the layers of metal dichalcogenide systems continues, it is clear that the interlayer region plays a critical role in determining the properties of these materials.

Most of the recent reviews cover the electronic and optical properties of TMDs and their heterostructures, as well as their optoelectronic applications [33,34,35,36,37]. The energy crisis and the need to use clean resources have urged researchers to pay attention to the design and construction of energy storage devices and energy production from renewable sources [38,39]. Among the mentioned devices, types of electrochemical batteries and solar cells have received more attention than others due to their extraordinary advantages. One of the most essential parts of batteries and solar cells is the material used in their electrode structure. Reviewing recent scientific sources confirms the attractiveness of introducing different materials for use in the electrode of these types of equipment. Oxides and sulfides of transition metals, as well as a combination of these materials, are attractive, inexpensive, and relatively stable options in catalysts, especially in energy [40,41,42,43,44,45]. Among the mentioned materials, TMDs are of great interest due to their very specific structure and extraordinary physical and chemical properties. In this review, the focus is on TMDs, mainly MoS_2_, used in the field of energy production and storage. This article describes the applications of these materials in electrochemical batteries and solar cells.

## 2. Transition Metal Dichalcogenides in Electrochemical Batteries

TMDs have gained significant attention in recent years for electrochemical applications. Their layered structure gives rise to several interesting electrochemical properties that make TMDs attractive for use in a variety of applications, including energy storage, energy conversion, sensing, catalysis, and electronics. One of the key aspects of the electrochemical behavior of TMDs is their high electrochemical stability. TMDs exhibit minimal degradation and corrosion in electrochemical environments, making them useful for applications in energy storage and conversion devices. This high electrochemical stability is due to the strong covalent bonds between the atoms in the layers and the large energy band gap. This combination of properties makes TMDs resistant to degradation and corrosion in electrochemical environments. TMDs such as molybdenum disulfide (MoS_2_), tungsten disulfide (WS_2_), and titanium disulfide (TiS_2_), have been used as materials for electrochemical energy storage applications, such as batteries and supercapacitors, due to their high surface area, good electrical conductivity, and high stability. In batteries, TMDs can be used as cathode materials because they can store and release a large amount of lithium ions, leading to high specific capacity. For example, MoS_2_ has a theoretical specific capacity of about 670 mAh g^−1^, which is higher than many other cathode materials used in lithium-ion batteries. Additionally, the layered structure of TMDs provides a large surface area for lithium-ion diffusion, which can improve battery performance [46]. 

Another important aspect of the electrochemical behavior of TMDs is their large surface area. TMDs have a large surface area compared to their volume, which enhances their electrochemical activity and makes them attractive for use in electrochemical sensing and catalysis applications. This large surface area is due to the layered structure of TMDs, which allows for a large number of atoms to be exposed at the surface. This large surface area increases the electrochemical activity of TMDs, making them attractive for use in applications where electrochemical reactions are important [46,47]. 

The start of the era of modern batteries, as energy storage devices, can be dated to the early 18th century when Alexander Volta introduced the voltaic pile [48]. Basically, batteries are composed of two electrodes separated by an electrolyte, with chemical energy stored in the active electrodes [49]. The chemical energy is directly converted to electrical energy through electrochemical redox reactions or charge-transfer reactions [50]. The way batteries work is that the reduction reactions that take place at the negative electrode produce electrons that through the external circuit reach the positive electrode where oxidation reactions take place [51].

There are different types of batteries, but they are generally divided into two main categories [52]: primary batteries, commonly known as non-rechargeable batteries, and secondary batteries which include rechargeable batteries. Primary batteries are used in small (portable) electrical devices and are generally non-rechargeable and recyclable after use. The most common are alkaline and zinc–carbon batteries with aqueous or non-aqueous electrolytes [53,54]. Secondary batteries are widely used in everyday life applications, like cell phones and electric vehicles, and include lithium-ion, sodium-ion, magnesium-ion, aluminum-ion, calcium-ion batteries with nickel-metal hydride, nickel–cadmium, and so on. These batteries are classified based on the material of their electrodes [55,56].

Higher efficiency, longer life, flexible design, and optimal energy density are the reasons for the high acceptance of lithium-ion batteries [57]. In commercialized lithium-ion batteries, graphite and Li^+^ have been used as anode and cathode, respectively, which have drawn attention to new materials owing to their low performance [58].

Today, many materials are used to improve battery performance. Among them, TMDs are widely exploited, especially in rechargeable secondary batteries. TMDs are abundant in natural minerals and have a high charge–discharge, long life, and a high oper-ating temperature range.

One of the main energy storage mechanisms of TMDs is lithium-ion intercalation. In this process, lithium ions are inserted into the TMD crystal structure, causing a change in its electrical properties, and allowing for the storage of electrical energy. The reversible intercalation of lithium ions also provides a high capacity for energy storage, making TMDs attractive for use in lithium-ion batteries. Another mechanism is the capacitive energy storage that takes advantage of the high electrical conductivity of TMDs. In this process, charge is stored on the surface of the TMD material, creating a double-layer capacitor. The high surface-to-volume ratio of TMDs results in a high capacitance, allowing for efficient energy storage. TMDs also show promise as supercapacitor materials due to their high charge storage capacity and excellent stability. The energy storage mechanism in supercapacitors is based on the rapid transfer of charge between the TMD and the electrolyte, which enables rapid charging and discharging [59].

In this review, we focus on the application of TMDs in batteries, such as lithium-ion and sodium-ion batteries, which are widely used in electric vehicles, energy storage bases, and smart networks. In this domain, the most used TMDs are MoS_2_ and FeS_2_.

### 2.1. MoS_2_, MoS_2_-Metal Oxides, and Other TMDs in Lithium-Ion Batteries

TMDs have a layered crystal structure consisting of a transition metal layer sandwiched between two chalcogenide layers. The chalcogenide atoms can be sulfur (S), selenium (Se), or tellurium (Te), while the transition metal atoms can be molybdenum (Mo), tungsten (W), or other transition metals. The most commonly studied TMDs are MoS_2_, WS_2_, and their derivatives. 

The layered structure of such TMDs is similar to graphite, with strong covalent bonds within each layer and weak van der Waals forces between the layers. This results in a material that is mechanically flexible, optically transparent, and has high surface area. The electronic properties of TMDs are highly dependent on the number of layers. When TMDs are in their two-dimensional form (i.e., with one or few layers), they often exhibit a direct band gap, which makes them attractive for optoelectronic applications. As the number of layers increases, the band gap typically becomes indirect, leading to changes in the material’s electronic properties.

To realize the full potential of TMD materials, a variety of synthetic techniques have been developed for their production. TMDs can be synthesized by mechanical exfoliation, chemical vapor deposition (CVD), solution-based methods, etc.

One of the most commonly used techniques for synthesizing TMDs is chemical vapor deposition (CVD). CVD involves the thermal decomposition of a precursor gas, which is introduced into a reaction chamber along with a substrate. The gas reacts on the substrate, forming a TMD material layer. CVD is a versatile technique that can be used to produce TMDs with different sizes, shapes, and properties [60]. 

Mechanical exfoliation is another technique that is used to produce TMDs. This technique involves peeling off individual layers of TMDs from a bulk crystal using scotch tape. The resulting flakes can be transferred to a substrate to form thin films. This technique is commonly used to produce high-quality TMDs for research purposes [61]. 

Hydrothermal and solvothermal synthesis are two other techniques used to synthesize TMDs. Hydrothermal synthesis involves reacting transition metal salts and chalcogenide salts in a high-pressure autoclave at elevated temperatures, while solvothermal synthesis involves reacting these salts in a solvent at high temperatures and pressures. Both of these techniques can produce TMDs with controlled sizes and shapes [62]. 

Electrochemical synthesis is a technique that involves electrochemically reducing transition metal ions and chalcogenide ions in a solution containing an electrolyte. This technique can produce TMDs with high purity and controlled morphology [63]. 

Ion exchange is another technique that is used to produce TMDs. This technique involves replacing the cations in a layered TMD material with other metal ions through ion exchange reactions. This technique can produce TMDs with unique properties, such as enhanced catalytic activity [64]. 

Thermal stability of TMDs is an important factor in their potential use in energy applications. The high thermal stability of TMDs makes them attractive for use in high-temperature environments and suggests that these materials have great potential for use in a range of energy-related applications. Thermal stability refers to a material’s ability to resist degradation or decomposition when exposed to high temperatures. For TMDs, which are being considered for use in high-temperature environments, high thermal stability is particularly important. Fortunately, TMDs have been found to exhibit high thermal stability, with many materials able to withstand temperatures of up to 700–800 °C without significant degradation [65]. 

For example, WS_2_ has a thermal stability of up to 900 °C in air and up to 1000 °C in vacuum. This high thermal stability makes WS_2_ a promising material for high-temperature applications such as thermoelectrics, which involve converting waste heat into electricity. Similarly, MoS_2_ has a thermal stability of up to 600–700 °C, making it suitable for use in catalysis and energy storage [66].

The high thermal stability of TMDs is due to the strong covalent bonding between the transition metal layer and the chalcogenide layers in the material. This bonding makes the material resistant to thermal degradation, allowing it to maintain its structural and electronic properties even at high temperatures [67]. Other TMDs, such as molybdenum diselenide (MoSe_2_) and tungsten diselenide (WSe_2_), have also been found to exhibit high thermal stability in the range of 500–700 °C. This suggests that TMDs have great potential for use in a range of high-temperature energy applications, including thermoelectrics, catalysis, and energy storage [68]. The measured room temperature thermal conductivity of TMDs such as MoS_2_, MoSe_2_, MoTe_2_, WS_2_, and WSe_2_ is around ∼154 W m^−1^ K^−1^, ∼70 W m^−1^ K^−1^, ∼77 W m^−1^ K^−1^, ∼262 W m^−1^ K^−1^, and ∼120 m^−1^ K^−1^, respectively [69].

The pure MoS_2_ is prepared by many physical and chemical methods such as chemical vapor deposition, thermal decomposition, and gas phase synthesis.

**Figure 1:**
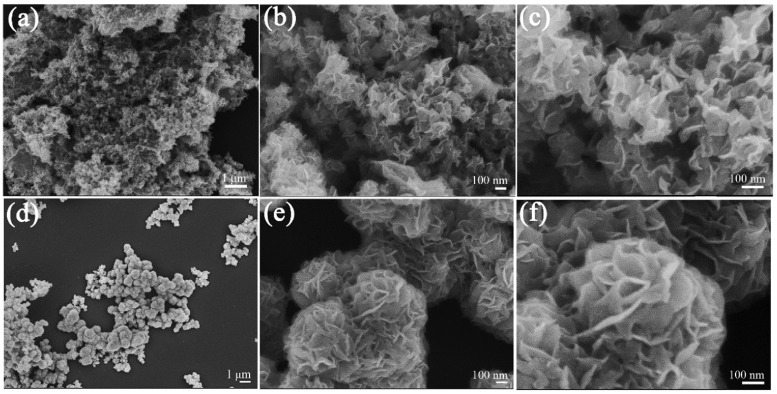
FESEM images of MoS_2_ nanosheets (**a**–**c**) and MoS_2_ nanoflowers (**d**–**f**) at different magnifications. Reprinted (adapted) with permission from Reference [74], Copyright (2019), American Chemical Society.

Structurally, MoS_2_ comes in two forms, nanosheets and nanoflowers, both of which are layered structures used in rechargeable lithium-ion batteries (Figure 1). A comparison shows that for MoS_2_ nanosheets the reversible discharge capacity is 589 mAh g^−1^ at 100 mA g^−1^ after 80 cycles while for MoS_2_ nanoflower it is 883 mAh g^−1^ at 400 mA g^−1^ after 30 cycles, indicating the importance of the material morphology on the performance of the electrode. The discharge capacity depends also on the synthesis methods. A reversible discharge capacity of ~850 mAh g^−1^ at 100 mA g^−1^ after 50 cycles has been found with MoS_2_ produced by the hydrothermal method. These results demonstrate that MoS_2_ suits fast charge–discharge needs [70,71,72,73].

Accumulation of MoS_2_ plates during lithium-ion battery cycles is a major problem. Therefore, oxides with suitable reversible discharge capacity were used to facilitate the penetration of the electrolyte between the MoS_2_ plates and separate the plates to prevent them from accumulating on top of each other. TiO_2_ is known as a suitable anodic material for lithium-ion batteries with suitable properties such as low cost, easy access, and environmental friendliness. There is also a strong synergistic effect between MoS_2_ and TiO_2_, which makes the transport of ions and electrons easier at the interface. For example, compounds such as hydrothermal plate MoS_2,_ coated with TiO_2_ tablet-shaped plates or TiO_2_ microsphere structure and MoS_2_ plates together have a high cyclic capacity of about 714–740 mAh g^−1^ after 150–200 cycles at 100 mA g^−1^. TiO_2_ has also been used as an interfacial bonder to create a strong bond between MoS_2_ and other compounds, allowing electrons to be easily transported across the electrode. In addition to TiO_2_, other metal oxides, such as Fe_3_O_4_ and SnO_2_, which have high capacities and are suitable for preparing electrode materials in the form of composites with MoS_2_, have also been reported. These composites showed good reversible discharge capacity [75,76,77,78,79,80].

### 2.2. MoS_2_—Carbon Composites in Lithium-Ion Batteries

#### 2.2.1. MoS_2_–Graphene Hybrid

Graphene is a carbon material with a large specific surface area, excellent electron mobility, high thermal conductivity, and a carbon framework with sp^2^ hybridization. Hence it is used as a matrix to support the anode or cathode in rechargeable lithium-ion batteries. There are different methods for preparing MoS_2_-graphene composite. These methods include hydrothermal liquid phase, chemical vapor deposition, and liquid phase with temperature correction. There are other easy fabrication methods such as heat-induced formation process that result in MoS_2_–graphene sheet composite with good efficiency of 552 mAh g^−1^ at a current density of 10 A g^−1^ after 7500 cycles [81]. The synergistic effect created from integration of MoS_2_ and graphene leads to enhanced lithium storage performance.

#### 2.2.2. MoS_2_–Amorphous Carbon Hybrid

These types of carbon materials are very inexpensive and are used in rechargeable lithium-ion batteries to increase the distance between MoS_2_ plates and achieve the desired conductivity. Use of amorphous carbon in the combination of MoS_2_–amorphous carbon showed relatively good results. So, this composite with suitable preparation methods can be used to form a uniform, inexpensive hybrid with optimal layout in rechargeable lithium-ion batteries [82,83,84].

#### 2.2.3. MoS_2_–Carbon Nanotube Hybrid

Carbon nanotubes are one of the carbon allotropes. Carbon nanotubes are divided into two categories: multi-walled and single-walled. Nanotubes have been noted for their excellent thermal conductivity, and good mechanical and electrical properties, and have a special place as a modifier in rechargeable lithium-ion batteries. 

**Figure 2:**
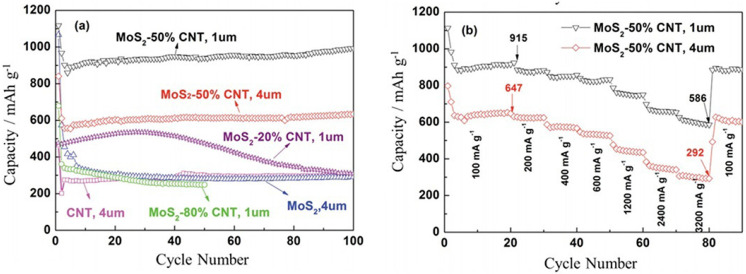
(**a**) Electrochemical performance of SWNT, MoS_2_, and MoS_2_–SWNT films between 0.01 and 3.0 V vs. Li/Li^+^ at a cycling rate of 100 mA g^−1^, and (**b**) rate capacities of MoS_2_–SWNT (50%) films with a thickness of 1 μm and 4 μm. Reprinted (adapted) with permission from Reference [89], Copyright (2013), Wiley.

**Figure 3:**
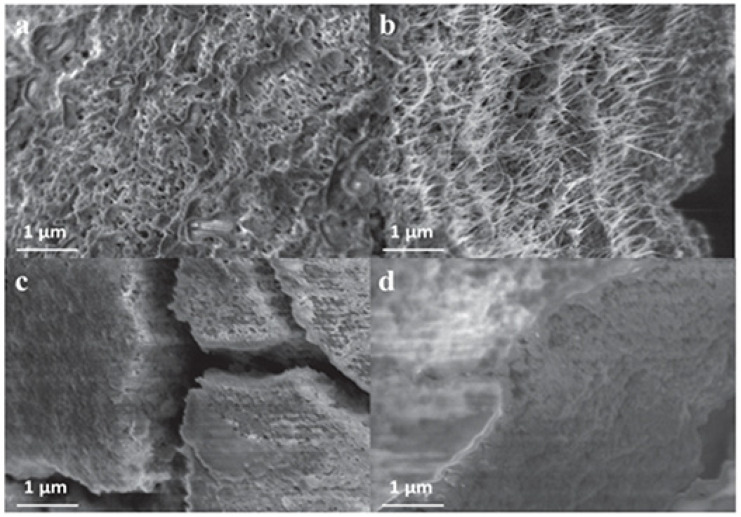
FESEM images of (**a**) MoS_2_–SWNT composite film, (**b**) cross-section of MoS_2_–SWNT composite film (**c**), pure MoS_2_ film electrode and (**d**) cross-section of pure MoS_2_ film electrode after cycling. Reprinted (adapted) with permission from Reference [89], Copyright (2013), Wiley.

The combination of carbon nanotubes with MoS_2_ has been conducted in different ways and has provided good results [85,86,87,88]. The composition of MoS_2_ and single-walled carbon nanotube through the liquid-phase exfoliation of MoS_2_ showed a strong electrochemical capacitance of 992 mAh g^−1^ after 10 cycles with retention of 82% of the initial capacitance of 1117 mAh g^−1^ (Figure 2). In addition, the morphology of MoS_2_–SWCNT thin film (Figure 3) retained structural impeccability after 100 cycles, while the MoS_2_ thin film without SWCNTs displays cracks, which indicate a mechanical degradation issue [89].

#### 2.2.4. MoS_2_–Polymer Hybrid

Conductive polymers are another material used in MoS_2_-based rechargeable batteries as molybdenum disulfide modifiers, for example combining a conductive polyaniline polymer with molybdenum disulfide. Of course, these conductive polymers have properties that limit their use in batteries based on TMDs. The limiting properties of these polymers are rigidity of the chains (which disrupts the movement of ions), high cost, insoluble, low permeability, and supply problems [90,91,92].

### 2.3. Other Layered TMDs in Lithium-Ion Batteries

Other layered TMDs used in rechargeable lithium-ion batteries include WS_2_, TiS_2_, and VS_2_. due to their high theoretical capacity and good power density [93,94,95,96].

WS_2_ is a promising material to use for the anode in lithium-ion batteries. The interlayer distance in WS_2_ is about 0.63 nm, which is greater than graphite with an interlayer distance of 0.32 nm and provides more paths for intercalation of Li+. Liu et al. prepared a sample of mesoporous WS_2_ by a vacuum-assisted impregnation method. To achieve a high surface area (115 m^2^ g^−1^) during a process, quantities of gases that were trapped in the internal pores were forced outwards. The high discharge capacity for mesoporous WS_2_ was obtained after 100 cycles with a discharge rate of 100 mA g^−1^ that was equal to 805 mA h g^−1^. Additionally, during discharge current of 10 mA g^−1^, it showed a high rate capacity of 503 mA h g^−1^ [97].

Bhandavat et al., with the help of acid exfoliation method and by chlorosulfonic acid, were able to convert bulk WS_2_ into few-layer two-dimensional WS_2_ sheets and achieve reversible capacity of 469 mA h g^−1^ at a discharge rate of 25 mA g^−1^ for lithium-ion batteries [98].

### 2.4. Layered TMDs in Rechargeable Sodium-Ion Batteries

Sodium-ion batteries are a more cost-effective method of energy storage used for large-scale energy storage applications. Applications for rechargeable sodium-ion batteries include energy storage bases, energy storage networks, and storage of renewable energy such as wind and solar power, in backup systems as a permanent power supply. Like lithium-ion batteries, TMDs such as MoS_2_ and TiS_2_ are widely used in sodium-ion batteries. As mentioned earlier, MoS_2_ and other TMDs have been used both in pure form and in composite form with carbon and non-carbon materials in sodium-ion batteries [99,100,101,102].

In general, the range of applications of MoS_2_ is reduced owing to inherent restrictions such as low electrical conductivity [99]. Hence, the applications of TMDs other than MoS_2_ to prepare TMD-based electrodes with excellent and reliable performance for use in batteries are highly regarded. A good example is MoSe_2_, which has a narrower band gap and larger interlayer spacing compared to MoS_2_, and these properties lead to better performance as anodes in sodium-ion batteries [103].

Similarly, another important member of the TMD family, which is MoTe_2_, with an interlayer distance of about 0.70 nm, can accommodate large-sized ions such as Na^+^ (0.35 nm) [104]. Moreover, Te has better electronic conductivity (2 × 10^2^ S m^−1^) than S (5 × 10^−13^ S m^−1^) and Se (1 × 10^−3^ S m^−1^), making MoTe_2_ a good anode material [104]. Indeed, MoTe_2_ as the anode in sodium-ion batteries has exhibited acceptable performance stability [105]. 

Overall, TMDs appear as promising electrode materials for electrochemical energy storage in rechargeable batteries.

### 2.5. Non-Layered TMDs in All Types of Rechargeable Batteries

Another category of TMDs includes non-layered structures, which are used as electrode materials in lithium-ion batteries as well as rechargeable sodium-ion batteries. Non-layered TMDs, which are in the category of sulfide minerals, are used as electrode materials in a variety of rechargeable batteries due to their properties such as cost-effectiveness, high capacity, low toxicity, availability, and suitable frequency.

One of the most common sulfide minerals in the semiconductor category is pyrite (FeS_2_), which has been used both as pure and in composite with carbon fiber, graphene oxide, nanotubes, and other materials in rechargeable lithium-ion and sodium-ion batteries [101].

CoS_2_ has a pyrite structure, and it can play the role of cathode in thermal batteries. It has also been used to replace pyrite in rechargeable batteries as an electrode material. This material is not yet widely used but has good potential for use in rechargeable batteries [106].

### 2.6. Storage Mechanisms

**Figure 4:**
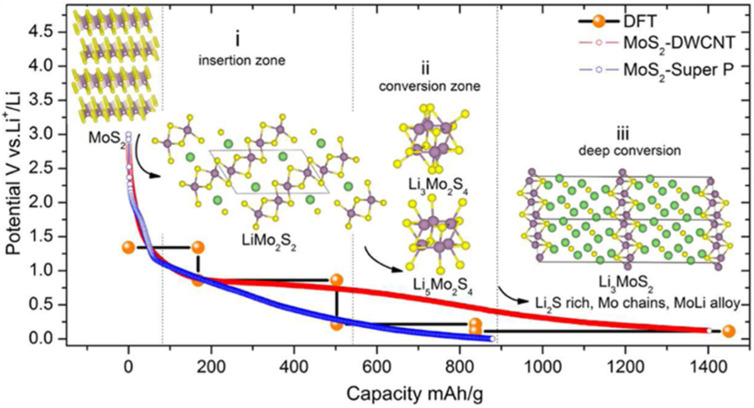
Li-ion transport mechanism of the MoS_2_ structure in lithium-ion batteries. Reprinted (adapted) with permission from Reference [112], Copyright (2016), American Chemical Society.

In rechargeable batteries, the storage mechanism of Li, Na, and K ions has been extensively studied using experimental methods and theoretical calculations (Figure 4). In most cases, alkali metals act likewise in charge storage owing to their similar physical properties [107,108,109]. Nevertheless, exacerbating the diffusion resistance and the increase of the volume of TMDs is possible based on the increase of the ionic radius from Li^+^ ion to K^+^ ion [110,111].

**Figure 5:**
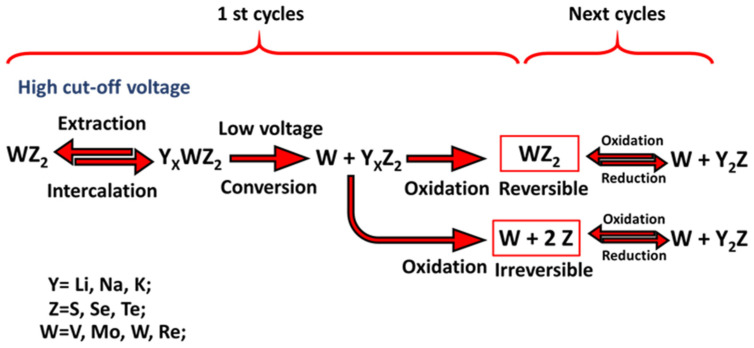
Schematic of electrochemical storage mechanisms for WZ_2_ in rechargeable batteries. Reprinted (adapted) with permission from Reference [115], Copyright (2020), Royal Society of Chemistry.

The cut-off voltage and the properties of TMDs are effective on the storage mechanism of rechargeable batteries (Figure 5). At high cut-off voltages of about 1 V, reversible intercalation and extraction reactions occur for all TMDs [113]. On the other hand, at low cut-off voltages of about 0.1 V, for some TMDs with Ti and Nb, reversible intercalation and extraction reactions can still be performed due to the interactions in the bond between groups (T = Ti, Nb) with (Z = S, Se, and Te), while the TMDs including (W = V, Mo, W, and Re) do not have the ability to act in these conditions [114]. After the first discharge process in rechargeable batteries, WZ_2_ (W = V, Mo, W, and Re) alone and a compound with formula Y2Z (Y = Li, Na, K and Z = S, Se, and Te) are formed in the conversion storage mechanism.

The dynamic properties of TMDs are effective on oxidation products [114]. After the initial charge, due to weak dynamic properties, (W = V, Mo, W, and Re) and (Z = S, Se, and Te) are irreversibly formed. In the next step, the remaining active material from the first charge step changes from the combined state between (W = V, Mo, W, and Re) and (Z = S, Se, and Te) to (Z = S, Se, and Te) only in the following cycles. The highest dynamic properties will cause complete reversible oxidation. Compound (W = V, Mo, W, and Re) and 2 (Z = S, Se, and Te) is formed during the first charge process and acts as an active material in the following cycles.

The intercalation mechanism of Li, Na, and K ions storage in (W = V, Mo, W, and Re) and 2 (S, Se, and Te) compounds has been established to be reversible, the intercalation mechanism of these compounds is similar to the assumed mechanism for graphite [116].

Until now, the intercalation mechanism of Li to the most famous member of the TMDs family, namely MoS_2_, has been studied by many researchers. In a 2012 study, to confirm the reversible reactions of commercial MoS_2_ at a cutoff voltage of 0.8 V, and to discover its crystal structure, Fang et al. used in situ X-ray diffraction analysis [116]. Based on numerous research studies that were conducted on the intercalation mechanism of Li, Na, and K ions in the structure of TMDs, especially MoS_2_, in general, the results of several analyses that were performed confirmed that the intercalation of Li ion in the structure is more than an emission of 4, also with the emission of ions within the MoS_2_ structural network, there undergoes a noticeable phase change. Alternatively, the results confirmed that the optimal cut-off voltage range to prevent the damage of MoS_2_ layered structure is in the range of 3 to 0.6 V [115].

## 3. Transition Metal Dichalcogenides in Solar Cells

All radiation in the electromagnetic spectrum, including sunlight, can be thought of in terms of photons, which carry certain amounts of energy that depend on the wavelength. To create an electron-hole pair, mainly the photons with energy greater than the band gap of a semiconductor contribute to the energy conversion process [117].

In semiconductors, which are the materials used in solar cells, there is a forbidden band or band gap that separates the valence and conduction bands. If the photon received by the semiconductor provides enough energy to the electron in the valence band to be transferred to the conduction band, a photovoltaic (PV) effect is possible. But if the band gap is large such that no electron can absorb light and make a transition to the conduction band, then this material will not have the necessary efficiency as a semiconductor. Therefore, materials with a large band gap have not been a good option in solar cells. Small-band gap materials are also not suitable, because the electron receives energy that transfers it to the higher levels of the conduction band. As there are lower energy levels in the conduction band, the electron scatters in transit to these levels, wasting energy in the form of heat. As a result, the most efficient conversion of light energy into electricity occurs when the received photon energy is very close to the band gap energy of the material in question [118].

Increasing the performance of a solar cell is possible by increasing the power conversion efficiency (PCE) of the cell, reducing production costs, and increasing the life of the cell. Therefore, researchers have studied different materials to obtain PV cells with better performance, cost-effectiveness, and safety [119].

Conventional silicon (Si) solar cells have a 95% share of the photovoltaic market due to their low-cost fabrication and reasonable PCE. What reduces the performance of ultra-thin and flexible Si solar cells is the fragile nature of Si, which makes it inefficient in applications such as aerospace, transportation, architecture, and wearable electronics because they require high power per weight [120].

A new generation of advanced solar cells such as Dye-Sensitized Solar Cells (DSSCs), Organic Solar Cells (OSCs), Quantum Dot Solar Cells (QDSC), and Perovskite Solar Cells (PSCs) has emerged. Researchers are currently attempting to replace silicon solar cells with a new generation of cells.

### 3.1. Perovskite Solar Cells (PSCs)

Among the different types of solar cells, PSCs have attracted a lot of attention due to their high conversion efficiency, flexibility, low cost, and construction by the roll-to-roll method. Market conquest by a new generation of PSCs is predicted soon to take place [121]. Perovskite solar cells are a type of photovoltaic (PV) technology that uses perovskite material as the light-absorbing layer to convert sunlight into electricity. The perovskite layer is typically composed of a mixture of organic and inorganic materials, such as lead halides and organic compounds. One of the advantages of perovskite solar cells is their high light-to-electricity conversion efficiency, which has improved dramatically over the last decade, reaching over 25% in some cases. They are also relatively easy and cheap to produce, compared to other types of PV technologies. However, perovskite solar cells still face some challenges, such as stability and toxicity issues. Lead is a toxic element and its use in perovskite solar cells raises environmental and health concerns. In addition, the perovskite material is prone to degradation under harsh environmental conditions, such as high temperature and humidity. Despite these challenges, perovskite solar cells have the potential to become a game-changer in the renewable energy sector. Researchers are working to develop more stable and environmentally friendly perovskite materials, and to improve the manufacturing process and long-term stability of perovskite solar cells. Hence, scientists are evaluating different materials for making different types of solar cells. The use of ultra-thin nanometer-based light absorbers in solar cells reduces material consumption, charge emission length, and recombination. The use of two-dimensional materials such as graphene has also been useful in some solar cells [122].

### 3.2. TMD Solar Cells

Solar cells based on TMDs have attracted significant attention due to their unique optoelectronic properties, such as high absorption coefficients and efficient charge separation [123,124]. This has led to the development of TMD-based photovoltaic devices with improved performance compared to traditional silicon-based solar cells.

However, the development of TMD-based solar cells is still in its early stages, and there are several technical challenges that need to be addressed, such as improving the efficiency and stability of the devices. Nevertheless, the potential benefits of TMDs in the field of photovoltaics make them a promising area of research and development.

Semiconductor TMDs such as WS_2_, WSe_2_, MoS_2_, MoSe_2_, MoTe_2_, and TaS_2_ have shown remarkable optical and mechanical properties with adjustable electronic properties and structural control capabilities. For instance, the MoX_2_ (X = S, Se, Te) series, based on different levels of stacking between the layers, can be observed in several phases and has a crossover from an indirect band gap to a direct band gap [125,126,127]. In general, the band gap of TMD materials is in the range of 1 to 2 eV, which is in good compliance with the solar spectrum and is comparable with the band gap of Si (1.1 eV), GaAs (1.4 eV), and CdTe (1.5 eV) [128]. Most single-layer TMDs have a direct band gap and have potential as high-performance photovoltaic applications due to their high optical absorption coefficient and interesting electrochemical properties. Therefore, material engineering methods such as forming Mo_1-x_W_x_S_2_ alloys, applying mechanical stress, and controlling the band gap and ionization potential, can be very efficient for preparing suitable materials [129,130].

Researchers have studied the different functions of two-dimensional TMDs in different types of solar cells and introduced the most efficient ones such as MoS_2_/RGO (reduced graphene oxide) nanocomposite, MoS_2_-CuS composite, and MoS_2_/FTO as materials with suitable performance in counter electrodes, with ITO/MoS_2_/Au, MoSe_2_/GaN, n-MoS_2_/i-SiO_2_/p-Si as an active layer, PEDOT:PSS/MoS_2_/perovskite/PCBM and n-MoS_2_/Al2O_3_/p-S as cavity transfer layer, MoS_2_/P_3_HT:PCBM/V_2_O_5_, and WS_2_/P_3_HT:PCBM/V_2_O_5_ as the electron transfer layer [128].

**Figure 6:**
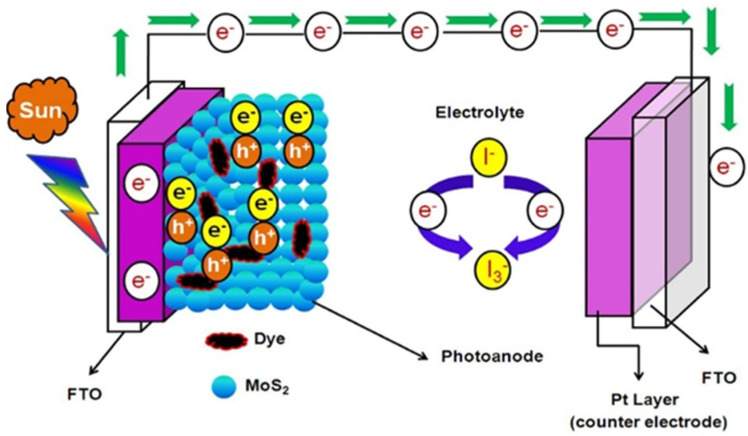
The schematic of MoS_2_/graphene nanocomposite-based photoanode for dye-sensitized solar cells (DSSCs). Reprinted (adapted) with permission from Reference [131], Copyright (2020), Elsevier.

It has been reported that MoS_2_ and WS_2_ as auxiliary electrode materials in dye-sensitized solar cells (Figure 6) have increased electrocatalytic activity due to their high electron mobility, good stability, and low cost. These materials have also been found to improve the light harvesting efficiency of DSSCs and to increase their stability compared to other semiconductors that are commonly used in these devices, such as TiO_2_.

The use of TMDs as counter electrodes and light absorbers has increased power conversion efficiency [132]. It has also been shown that it is possible to increase the efficiency of the solar cell by using TMDs as the cavity carrier layer and by optimizing the layer thickness. In this regard, various combinations of these materials with graphene, graphene oxide, rGO, WS_2_, MoS_2_ as a hole transport layer (HTL), and counter electrode (CE) materials in PV cells have been investigated [133].

### 3.3. Schottky Junction of Solar Cells

A Schottky junction is a type of metal-semiconductor junction commonly used in solar cells. It is formed by metals, such as aluminum or platinum, coming into contact with a semiconductor material, such as silicon. The junction is named after Walter H. Schottky, who first described it in the late 1920s. The use of Schottky junctions in solar cells provides several advantages. For one, they enable low-resistance electrical contacts, which help to increase the efficiency of the solar cell. They can also provide a low reverse-bias leakage current, which helps to reduce power losses in the cell. In addition, Schottky junctions can be made with a wide range of metal materials, which makes them versatile for use in various types of solar cells.

### 3.4. TMDs in Schottky Junction Solar Cells

In Schottky junction solar cells, two-dimensional TMDs are used in connection with metals or graphene to form interfaces for the separation of photoinduced charge carriers [122]. At the Schottky junction, a sudden potential difference occurs, the Schottky barrier, due to the difference in energy levels between the metal Fermi level and the conduction or the valence band of the TMD [134].

In Au/MoS_2_-based Schottky junction solar cells for 220 nm MoS_2_ stacks, a power conversion efficiency equal to 1.8% was reported [135]. In another study, a WS_2_/graphene-based solar cell in which WS_2_ with a thickness of 37 nm in contact with multilayer graphene was able to produce a PCE equal to 3.3% [136]. Monolayer TMDs absorb 5 to 10 percent of the sunlight which is higher than GaAs and Si absorbance of the same thickness (less than one nanometer) [137].

Using ultra-thin/two-dimensional MoS_2_ nanosheets as an effective hole extraction layer in organic solar cells, researchers achieved a relatively high PCE of 8.11% and by a graphene/MoS_2_/n-Si based photovoltaic device gained a PCE of 11.1% [138].

The presence of non-covalent interaction and the absence of dangling bonds on the surfaces of two-dimensional TMD is the reason for their high resistance to reaction with other chemical species. In addition, layered TMD non-bonded surfaces enable the use of heterogeneous structures, without lattice matching limitations, so called van der Waals heterostructures, which offer many options for designing TMD photovoltaics. Therefore, nanosheets of different materials are expected to be used in organic solar cells to improve stability [139].

According to realistic balance models, in very thin TMD single-junction solar cells a PCE close to 27% can be obtained. Despite this prediction, the real PCE of TMD solar cells is usually no more than 2% which is mainly due to the damage done to the TMDs. Pinning of the Fermi level of the metals as well as doping by non-practical methods such as diffusion and ion implants and fabricating on a flexible substrate can cause contamination or damage to TMD heterojunctions and reduce performance.

One of the factors that significantly improved the performance of WSe_2_ solar cells which led to a record of 5.1% PCE and a record of power of 4.4 W g^−1^, was the reduction or elimination of Fermi level pinning by adopting a metal transfer method. Additionally, putting a very thin intermediate layer of transparent graphene in the TMD-metal junction effectively improved the performance of the target cell.

The use of MoO_x_ coating with an anti-reflective effect for doping and a direct transfer method for fabrication as a clean and harmless procedure for the light-weight flexible polyimide substrates are other effective factors contributing to the record of PCE [140,141].

Moreover, the use of TMD-compatible doping methods to form a single p-n junction, such as surface charge transfer and fixed charge doping through metal oxides, plasma doping, or electrostatic doping, has significantly improved the performance of solar cells. The highest PCE is 2.8% in plasma-doped MoS_2_ thin-film single-bonded TMDs and 6.3% in MoSe_2_ solar cells electrostatically doped. TMD-based solar cells have been reported to suffer from relatively low external quantum efficiency (EQE) and low open-circuit voltage due to the design and construction of the device that is not optimized [142].

The band energy of the WSe_2_ is approximately 1.3 eV. Extremely high electron-hole mobility and high absorption coefficient (10^5^ cm^−1^ at 780 nm compared to silicon 10^3^ cm^−1^) make it one of the most promising TMD materials for high-performance photovoltaic applications.

Most work in TMD photovoltaics has been concentrated on the monolayer type due to the direct nature of the band gap, but monolayer TMDs can only absorb 10% of the incoming light. Although the absorption can be increased by using intensifiers, the use of multilayer WSe_2_ has been very effective.

**Figure 7:**
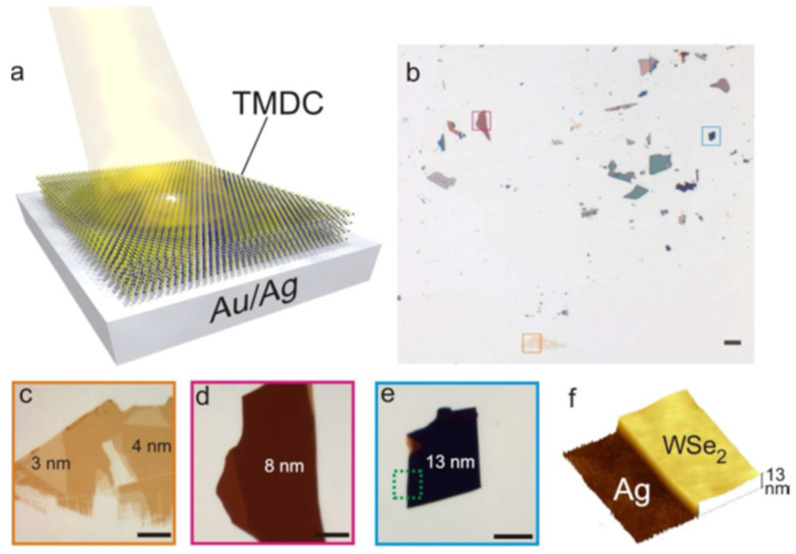
Absorbing TMD on metals: (**a**) Schematic diagram of a thin, multilayer TMDC film on an Au/Ag back reflecting substrate. (**b**) Low magnification optical micrograph of exfoliated WSe_2_ flakes on template stripped Ag substrate. (Scale bar = 50 μm) (**c**–**e**) High magnification micrographs of yellow, red, and blue square regions on (**b**) respectively with increasing flake thickness from (**c**) to (**e**). The sharp blue shift in color and rising contrast with increasing thickness can be seen (Scale bar = 10 μm). (**f**) AFM topography of the flake region in (**e**). Reprinted (adapted) with permission from Reference [143], Copyright (2016), American Chemical Society.

Jariwala et al. showed that a suitable photonic design for WSe_2_ multilayer shells and the use of a silverback reflector (Figure 7) can maximize absorption through Fabry–Perot effects to achieve an excellent absorption of over 90% with a thickness of approximately 15 nm [143].

Went et al. for investigation of the weakness of low open-circuit voltage in TMD-based solar cells, introduced a new metallization method for the junction line between the TMD absorber layer and metal, showing that gold transferred to the 16 nm WS_2_ layer could create a large Schottky barrier. This results in a PCE of 0.46% and an open-circuit voltage of 0.256 V at 1.5 AM [134].

**Figure 8:**
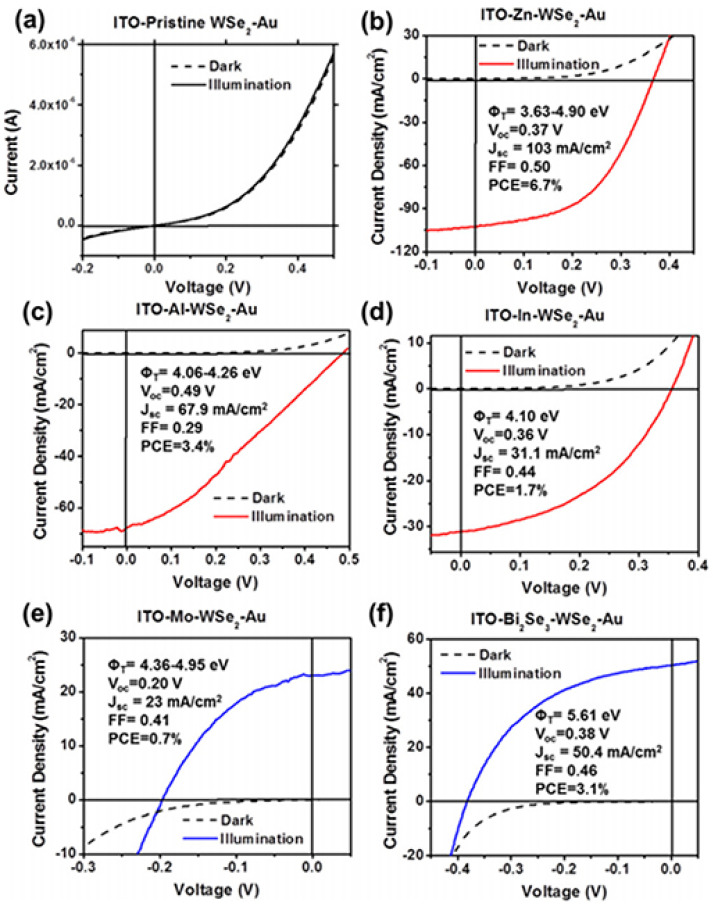
Photovoltaic characteristics of representative WSe_2_ PV devices coated with various top metals, including (**b**) Zn, (**c**) Al, (**d**) In, and (**e**) Mo, as well as (**f**) semimetal Bi_2_Se_3_ nanocrystals. (**a**) Result from a control device with no top metal coating. Reprinted (adapted) with permission from Reference [144], Copyright (2015), AIP.

Wi and colleagues also studied various metals in contact with WSe_2_ and showed that the high Schottky barrier between the zinc joints and the 100 nm layer of WSe_2_ (Figure 8) can increase the open-circuit voltage by 0.35 volts [144].

However, advances have been made in optimizing the design and fabrication of TMD-based solar cells, such as the WSe_2_ Schottky junction solar cell, which has a power conversion efficiency of 0.6–0.8% at 532 nm. However, its value is still far from the expected value of 20–22%. To meet this expectation, these devices need to be designed and built using the most advanced electronic transmission, doping, and deactivation technologies [145].

Scientists have recently investigated the effects of Al_2_O_3_ on activation on the properties of Pt/WSe_2_ vertical Schottky junction solar cells. Using WSe_2_ multilayer as an adsorbent layer with a thickness of 150–80 nm, as the optimal thickness for photovoltaic performance and by introducing a surface deactivation technique, they have shown that it improves short circuits due to the improvement in EQE. They have reported EQE of more than 50% at wavelengths below 600 nm, which depends on the deactivation of the trap modes, the effects of the anti-reflective coating, and the reorientation of the carrier due to doping.

They also showed that these deactivation effects of Al_2_O_3_ improved the level of the optical current collector and suggested surface deactivation techniques as an easy way to adjust the performance of an electronic TMD optical device [146].

## 4. Conclusions

Transition metal dichalcogenides are an attractive option for researchers as unrivaled semiconductors in various fields of nanoscience and electrochemistry. We examined some applications of these materials in the field of energy production and storage. We briefly mentioned the generalities of these materials and the recent studies on the mechanisms of energy production and storage along with the applications in energy storage and production equipment such as electrochemical batteries and solar cells. It has been shown that TMDs—despite some shortcomings and problems due to their high-speed performance, long-term cycles, and high capacity in lithium-ion and sodium-ion batteries and generally other rechargeable secondary batteries such as zinc-ion, magnesium-ion, aluminum-ion, calcium-ion, and potassium-ion—can be used as suitable electrode materials in the future. In addition, TMDs have shown potential for use in the field of solar energy conversion, specifically in the development of photovoltaic devices. The unique electronic and optical properties of TMDs, such as their two-dimensional structure, high absorption coefficients, and strong light-matter interactions, make them promising for use as active materials in solar cells. TMDs have been demonstrated to have a high light conversion efficiency and are able to absorb a wide range of light frequencies, making them an attractive material for the design of next-generation solar cells with improved performance. Furthermore, TMDs have the potential to significantly reduce the cost of solar cell production by enabling the use of low-cost and scalable fabrication methods. Therefore, the use of TMDs in solar cells has the potential to greatly improve the efficiency and affordability of solar energy conversion, making them an important area of research and development for the advancement of the renewable energy industry.

## Data Availability

The data reported in this review can be found in the listed references.
